# Digital Monitoring of Anemia Control Measures in a District Using Anemia Mukt Bharat Health Management Information System Indicators

**DOI:** 10.7759/cureus.75248

**Published:** 2024-12-06

**Authors:** Surabhi Puri, Kapil Yadav, Shashi Kant, Sanjay Rai, William Joe

**Affiliations:** 1 Centre for Community Medicine, All India Institute of Medical Sciences, New Delhi, New Delhi, IND; 2 Centre for Population Research, Institute of Economic Growth, Delhi University, New Delhi, IND

**Keywords:** anemia, anemia mukt bharat, digital monitoring, health management information system, india

## Abstract

Introduction: Anemia is a severe public health problem in India, affecting more than 50% of individuals across most age groups. The Anemia Mukt Bharat (AMB) program, with a target of a three-percentage point reduction in anemia prevalence per year, developed a monitoring mechanism based on a set of 18 indicators and six key performance indicators (KPIs) derived from routine reporting in the Health Management Information System (HMIS). The study’s objective was to assess the status of anemia control measures in the district of Faridabad, Haryana, India, using AMB HMIS indicators from April 2018 to March 2019.

Methods: A cross-sectional study was conducted in the district of Faridabad, Haryana, from May 1 to June 15, 2019 (reference period: April 2018 to March 2019). The status of existing activities for anemia control was assessed by documentation of HMIS indicators identified by the AMB program for routine monitoring. Sources of data were HMIS, program reports, and district annual reports. Denominators provided by the AMB program were used for ascertaining proportions and the AMB index. A critical review of the generated data and a description of the collection and reporting process were done.

Results: Activities in place to control anemia were prophylactic iron and folic acid (IFA) supplementation and deworming to 6-59-month children, adolescents, and pregnant and lactating women. For children five to nine years of age, IFA was not provided in the district, but deworming was done. The coverage of IFA prophylaxis in children 6-59 months was 22.5% and 85.1% in adolescents. The 180-day IFA supplementation in pregnant women was 33.1%. The IFA stock status was not captured in the district. The AMB index for district Faridabad (April 2018 to March 2019) was 35.2. Gaps in understanding data elements and mismatches between AMB denominator data and census forecasts limited the data's accuracy.

Conclusions: Overall, the status of anemia control measures assessed by HMIS indicators was unsatisfactory in the study district. Number-based reporting and issues with data quality limited the use of data for decision-making. Periodic evaluations of anemia control measures at the district level may be required to achieve the targets set by the AMB program.

## Introduction

Anemia is a crucial health concern worldwide. The global burden of anemia is high, affecting 24.3% of the world’s population [1]. Developing countries are severely affected and are major contributors to the global burden [2]. Consequences of anemia range from behavioral delay, decreased cognitive development, and increased susceptibility to infections in children to an elevated risk of poor feto-maternal outcomes such as preterm delivery and post-partum hemorrhage [3-9].

India’s battle against anemia has been ongoing since 1970 when the National Nutritional Anemia Prophylaxis Programme was adopted. Despite these efforts, anemia is a severe public health concern [10], with more than half of the population across all age groups affected [11]. The recent Comprehensive National Nutritional Survey (2016-2018) revealed that 41% of pre-schoolers, 24% of school-age children, and 28% of adolescents are anemic, with a high prevalence of iron deficiency [12].

The persistent burden of anemia in India over the past few decades has necessitated a fresh approach to its control. This led to the inception of the Anemia Mukt Bharat (AMB) program in 2018, with an ambitious goal of reducing anemia prevalence by three percentage points annually [13]. The comprehensive strategy of the AMB program focuses on prophylactic interventions, point-of-care diagnosis, and treatment of all causes of anemia. It also incorporates a robust monitoring system, with district-wise quarterly reporting of 18 anemia-related indicators and six key performance indicators (KPIs) derived from routine reporting in the Health Management Information System (HMIS) on the AMB online dashboard.

District-wise reporting aids in identifying the critical programmatic factors affecting the implementation of control activities and better prioritization of anemia control interventions, considering the uniformity of physical infrastructure, operating guidelines, and logistics across a district. Besides upstream reporting of the indicators, values identify problem areas in the district to guide decision-making. This requires quality data for the correct interpretation of the reported values. This study was conducted to assess the status of the anemia control measures being implemented in district Faridabad, Haryana, using AMB HMIS indicators for a year period of April 2018-March 2019.

## Materials and methods

This was a cross-sectional study conducted in district Faridabad, one of the 22 districts in Haryana [14]. The Faridabad district had a population of 1,809,733 (Census 2011), predominantly urban (about 80%). Haryana had a computerized HMIS, where the data were entered online at a health facility starting from a primary health center and could be accessed at both district and state levels. The study was conducted from May 1 to June 15, 2019. The reference period for the study was April 2018-March 2019. The status of the existing activities undertaken for anemia was assessed by documenting the HMIS indicators identified by the AMB program to be used for routine monitoring. Program officers at the district hospital were requested to provide data pertaining to the HMIS indicators. Sources for the data were HMIS, program reports, and district annual reports. The online HMIS portal was accessed with the help of the District Monitoring and Evaluation Officer. The process of obtaining and reporting the indicators was described, along with a comment on the quality of the generated data. This was done by interviewing the district monitoring and evaluation officer, program officers - National Health Mission (NHM) and school health, the medical officer-in-charge, and auxiliary nurse midwives (ANMs) of conveniently selected primary facilities (two each in urban and rural areas). Denominators available on the AMB Dashboard were used to recalculate the coverage rates recommended by the AMB program for routine monitoring. These denominator values were compared with estimates calculated by applying population proportions for the state of Haryana (Census 2011) to Faridabad.

For the current study, anemia control measures were defined as any activities undertaken to reduce the burden of anemia. Data quality refers to the extent to which data measures what it is intended to measure. Accuracy or reliability refers to the correctness of the data collected, specifically regarding the actual number of services delivered or health events conducted. Reasons for poor data accuracy were explored in the following domains: gaps in understanding of data definitions and collection methods, data recording and data entry errors, and systemic errors-logical errors embedded in the system that remain unless underlying systemic issues are corrected-and misreporting.

AMB describes 18 HMIS indicators to monitor activities in place to control anemia [13]. The indicators were grouped according to the beneficiary group, which covered children 6-59-month olds, children 5-9 years of age, adolescents 10-19 years, pregnant women, and lactating women. Each group had coverage indicators for prophylactic iron and folic acid (IFA) supplementation and deworming, with an additional indicator of the proportion of anemic individuals among pregnant women tested. AMB identified a few of these indicators as key performance indicators [13]. An AMB scorecard was prepared by calculating the mean coverage of IFA supplementation across these selected target beneficiaries. These were used to score each district's performance, ranging from zero (worst) to 100 (best).

Statistical analysis: Data were extracted from existing program-related records whenever unavailable in the district HMIS. Percentages were calculated from data collected by record review, HMIS, and AMB dashboard, and the indicators were computed.

Ethical approval was obtained from the Institute Ethics Committee of All India Institute of Medical Sciences, New Delhi, India, and permission to conduct the study was also obtained from the district's Chief Medical Officer. Study tools were pretested in a different district of Haryana, and the learnings were incorporated into the final tools used.

## Results

**Figure 1:**
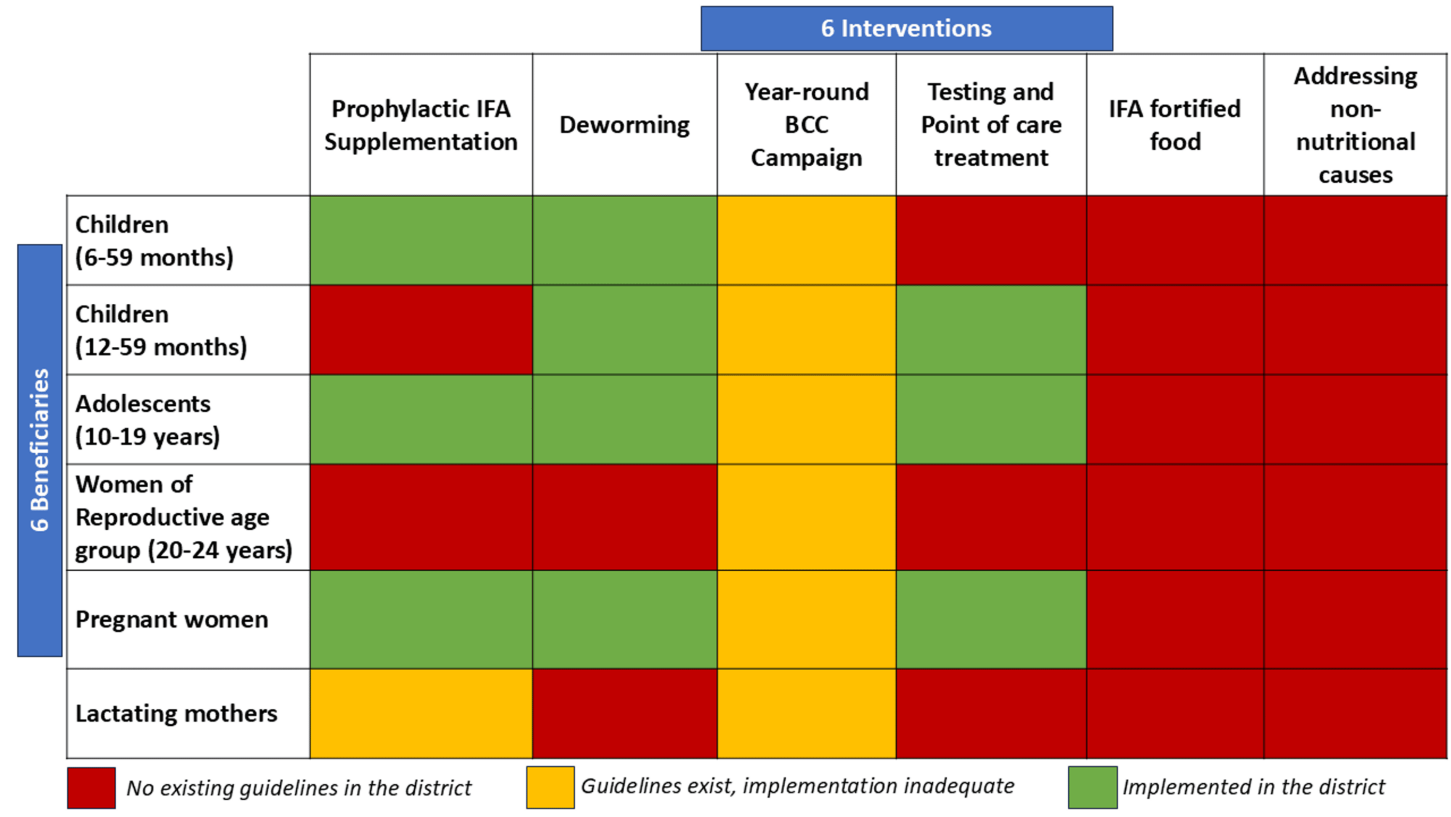
6x6 Matrix mapping the implementation status of the interventions for anemia control across different beneficiary groups in the district (April 2018-March 2019)

The AMB program was being rolled out in the district of Faridabad at the time of the current study. The activities undertaken in the district of Faridabad to control anemia (Figure 1) were prophylactic supplementation with adult IFA tablets in pregnant females and weekly IFA supplementation among adolescents in schools and Anganwadis. In the Faridabad district, supplementation was also provided to 6-59-month children with IFA syrup, under the Micronutrient Supplementation Programme, biannually along with vitamin A syrup. Point-of-care testing and treatment of anemia were done among OPD attendees and pregnant women. Deworming with albendazole was another control measure in the district. Biannual deworming on National Deworming Day was supplemented with routine field and OPD-based.

Process of reporting HMIS indicators

In the Faridabad district, health facilities routinely reported monthly HMIS indicator data. The ANM at each subcentre manually recorded these numerators, which the information assistant entered online at the facility level. The District Monitoring and Evaluation Officer (DMEO) accessed these data through the online HMIS portal, calculated indicator percentages for the district, and reported these to the state via email. The state could also directly access numerator data in HMIS.

Definition of data elements

Precise definitions of study elements are essential for accurately interpreting indicators. AMB provided definitions for all indicators [15], including their data sources, which closely matched those in the HMIS Service Provider’s Manual, except for elements 4.3 and 4.4. For these elements, only new cases of anemia and severe anemia were to be reported, whereas the HMIS manual included all new and existing cases. Definitions for data on IFA stock status were not provided in either source.

AMB HMIS Indicators for Faridabad (April 2018-March 2019)

The AMB HMIS indicators for Faridabad from April 2018 to March 2019 were compiled by accessing the district HMIS, district annual reports, and program reports (Table 1). The district HMIS did not report percentages; only number-based reporting existed. The only denominator value in HMIS was the number of registered pregnant women. The AMB Dashboard provided district- and state-wise denominator values based on population predictions to facilitate percentage calculation.

**Table 1:** Status of anemia control measures in the Faridabad district using AMB HMIS indicators (April 2018-March 2019) using the denominators provided on the AMB Dashboard for each beneficiary group *As reported by the National Deworming Day report (includes both government and private schools)

HMIS data element no.	Indicator	Numbers reported in the HMIS at the district level	Numbers reported to the State (percentage if reported)	Numerator/AMB denominator (percentage)
6-59-month children				
9.9	Percentage of children 6-59 months provided 8-10 doses (1 mL) of Iron and folic acid (IFA) syrup (biweekly)	39,673	39,673	39,673 / 176,614 (22.5)
9.10	Percentage of children 12-59 months provided Albendazole	62,236	62,236	62,236 / 156,990 (39.6)
Children 5-9 years				
23.1	Percentage of children covered under WIFS JUNIOR (5-9 years) provided 4-5 iron and folic acid (IFA) tablets in schools	Not given in Haryana	Not given in Haryana	-
23.2	Percentage of school children (5-9 years) provided albendazole at schools	Not reported in HMIS	158,427*	158,427*/ 44,004 (360)
23.3	Percentage of out-of-school children (5-9 years) given 4-5 iron and folic acid (IFA) tablets at Anganwadi Centres	Not given in Haryana	Not given in Haryana	-
23.4	Percentage of out of school children (5-9 years) provided albendazole at Anganwadi Centres	Not reported in HMIS	Not reported	-
Adolescents 10-19 years				
22.1.1	Percentage of adolescents (6-12 class) provided 4 iron and folic acid (IFA) tablets in schools	47,558	47,558	47,558 / 55,867 (85.1)
22.1.1.a	Percentage of adolescent girls (6-12 class) provided 4 iron and folic acid (IFA) tablets in schools	27,066	27,066	27,066 / 30,780 (87.9)
22.1.1.b	Percentage of adolescent boys (6-12 class) provided 4 iron and folic acid (IFA) tablets in schools	20,492	20,492	20,492 / 25,087 (81.7)
22.1.2	Percentage of (6-12 class) provided albendazole in schools	Not reported in HMIS	102,250*	102,250* /55,867 (183.0)
22.1.2.a	Percentage of adolescent girls (6-12 class) provided albendazole in schools	Not reported in HMIS	53,130*	53,130* /30 780 (172.6)
22.1.2.b	Percentage of adolescent boys (6-12 class) provided albendazole in schools	Not reported in HMIS	49,120*	49,120* / 25,087 (195.8)
22.1.3	Percentage of out-of-school adolescent girls 10-19 years provided 4 iron and folic acid (IFA) tablets at Anganwadi Centres	3574	3574	3,574 / 3,600 (99.3)
22.1.4	Percentage of out-of-school adolescent girls 10-19 years provided albendazole at Anganwadi centres	Not reported in HMIS	Not reported	-
Pregnant women				
1.2.4	Percentage of pregnant women given 180 iron and folic acid tablets	20,949	20,949 / 61,909 (33.8)	20,949 / 63,336 (33.1)
1.2.6	Percentage of pregnant women given 1 albendazole tablet after 1^st^ trimester	25,732	25,732 / 61,909 (41.6)	25,732 / 63,336 (40.6)
1.4.2	Percentage of pregnant women having Hb < 11 (tested cases) (7.1 – 10.9)	39,428	39428 / 61,909 (63.7)	39,428 / 63,336 (62.3)
1.4.3	Percentage of pregnant women having Hb < 7 (tested cases)	1,411	1,411 / 61,909 (2.3)	1,411 / 63,336 (2.2)
1.4.4	Percentage of pregnant women having severe anemia (Hb < 7) treated	1,360	1,360 / 61,909 (2.2)	1,360 / 63,336 (2.1)
Lactating female				
6.3	Percentage of mothers provided full course of 180 Iron and Folic Acid tablets after delivery	12,719	12,719	12,719 / 41,095 (31.0)

Children 6-59 months: For the first beneficiary age group of 6-59 months, HMIS captured two activities for anemia control: biweekly supplementation with IFI syrup and deworming with albendazole. The percentage of children 6-59 months provided 8-10 doses (1 mL) of IFA syrup (biweekly), calculated using the numerator reported in HMIS and the AMB denominator, was 22.5% (39,673/176,614).

Data on deworming coverage with albendazole were available in HMIS and National Deworming Day program reports. As per HMIS, the number of children (12-59 months) administered albendazole was 62,236 (39.6% (62,236/156,990) using the AMB denominator). These data were not reported in percentages to the state for this age group. For the given study period (April 2018-March 2019), only one round of National Deworming Day occurred in August 2018. The report claimed to have covered 191,466 beneficiaries in this age group. The calculated coverage using the AMB denominator for 12-59 months children was 122.0% (191,466/156,990).

Children five to nine years: For the age group of five to nine years, weekly IFA supplementation and deworming were expected to occur. However, the state of Haryana had not implemented weekly IFA supplementation (WIFS Junior) when this study was conducted. HMIS did not report any data for this section of AMB HMIS indicators. According to the National Deworming Day report, 158,427 children (five to nine years) were dewormed. However, the denominator for the same was not available at the district level. Using the AMB denominator, this coverage was 360% (1,58,427/44,004), flagging an issue with data obtained for this indicator.

Adolescents 10-19 years: To control anemia in the adolescent population of 10-19 years, weekly IFA supplementation and biannual deworming with albendazole were implemented. Using the AMB denominator, the Weekly Iron and Folic Acid program covered 85.1% (47,558/55,867) of its beneficiaries in the given age group. WIFS supplementation also covered 99.3% (3,574/3,600) of the out-of-school adolescent girls.

Deworming data for this age group were not reported in HMIS but were available in the NDD reports. Albendazole coverage was way above 100%, using numbers in NDD program reports as numerators and AMB denominators.

Pregnant women: As per HMIS data, the coverage of 180 IFA tablets provided to pregnant women was relatively low, at 33.1% (20,949/60,909). The values obtained for indicators dedicated to pregnant women were comparable with those obtained using AMB denominators (the difference was not more than 1%).

Lactating female: The percentage of lactating females receiving 180 IFA tablets was not reported to the state for the assessment period. However, the number of lactating females receiving 180 IFA tablets was reported in HMIS. Using live births (from HMIS) as the denominator, the calculated percentage of lactating females receiving 180 IFA tablets was 28.0%, comparable to the 31% obtained using AMB denominators.

Stock status: The consolidated district report did not provide data on any IFA formulation stock status, and the fields reporting this information were blank.

HMIS in the district followed number-based reporting. Indicator reporting in the form of percentages to the state via email was done majorly for the pregnant women beneficiary group. When calculated for other beneficiary groups, percentages were erroneous due to the unavailability of denominator values in HMIS.

A data element indicating diagnostic services for anemia - 1.4.1 in HMIS, giving the number of pregnant women tested for hemoglobin (Hb) 4 or more than four times for respective ANCs - was available in the HMIS but not included in the AMB HMIS indicators. The percentage of pregnant women tested for Hb 4 or more than four times for respective ANCs was 67.2% for the Faridabad district.

**Figure 2:**
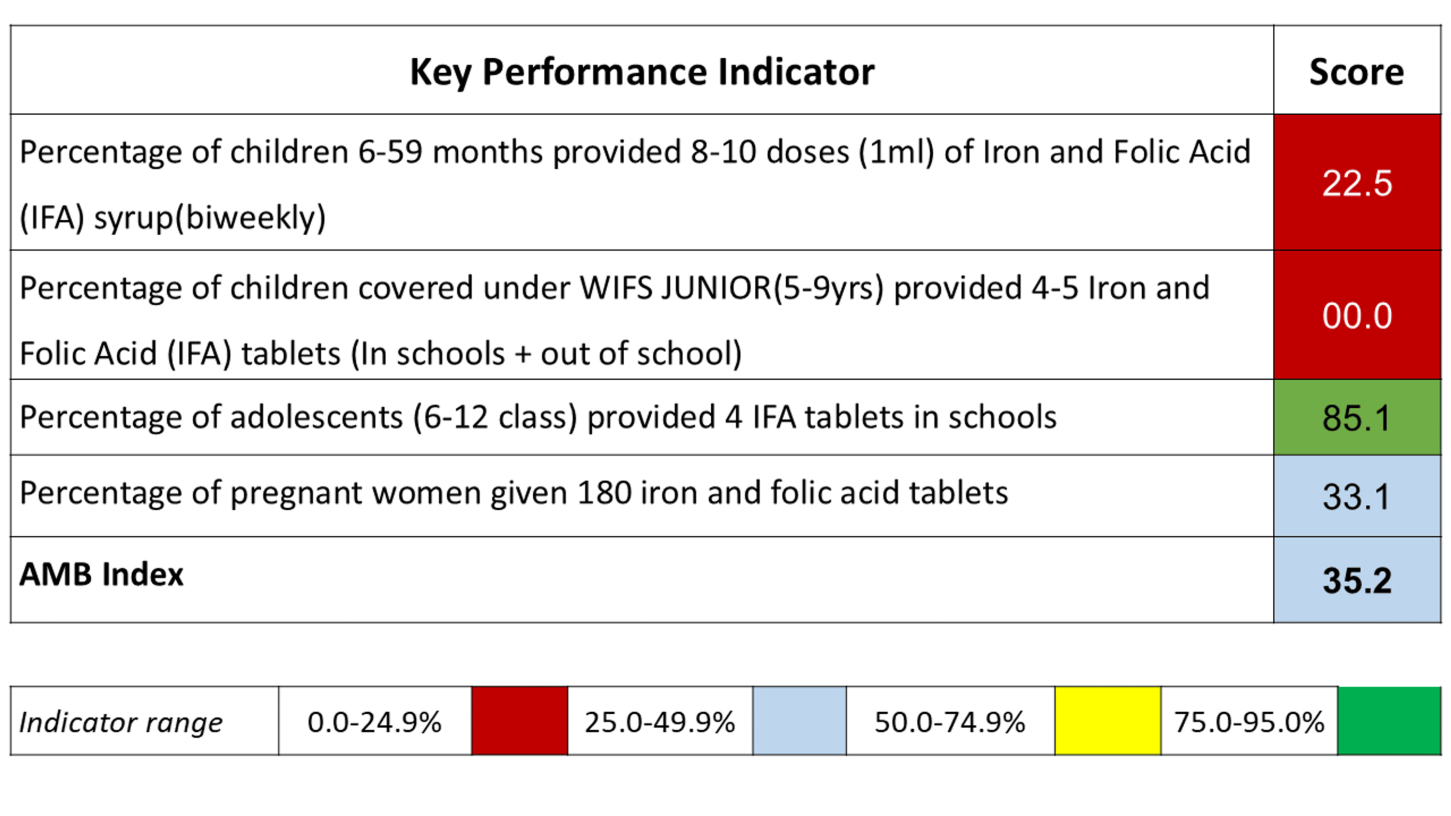
Anemia Mukt Bharat scorecard for the Faridabad district (April 2018-March 2019) The score mentioned against each KPI is the coverage (percentage) of IFA supplementation in that beneficiary group (using AMB denominators).

An AMB report card was prepared based on the district's KPIs. The AMB index for the Faridabad district was 35.2 (Figure 2). The report card highlighted gaps in implementing prophylactic IFA supplementation across most beneficiary groups.

Some of the values seemed erroneous when calculating indicator percentages. Hence, the AMB denominator values were compared with denominator values estimated from the census. The denominator values for children 5-9 years of age and adolescents 10-19 years of age seemed to be underestimated by AMB (6.9 and 13.72 percentage point difference, respectively) (Table 2). The percentage of adolescents (6-12 class) provided four IFA tablets in school, using the denominator estimated from the census, reduced from 85.1% to 14.1%, which would bring the AMB index by half to 17.6.

**Table 2:** Comparison of denominators for AMB indicators in HMIS, AMB dashboard, and Census estimates The data have been represented as N (%). *Estimated by applying the proportion of the total population in the desired age group for the state of Haryana as per Census 2011 to the population of the Faridabad district projected for the year 2018. **School-going children were estimated by applying the gross enrolment rate to the projected population (for the year 2018) of district Faridabad [16].

Denominators	HMIS	AMB Denominators (Percentage of total population: A)	Census estimated denominators* (Percentage of total population: B)	Percentage point difference (B-A)
Children 6-59 months	Not reported	176,614 (8.64)	173,706.1 (8.49)	-0.14
Children 12-59 months	Not reported	156,990 (7.68)	154,064.8 (7.53)	-0.14
Children 5-9 years in school**	Not reported	44,004 (2.15)	185,052.1 (9.05)	6.90
Adolescents 10-19 years in school**	Not reported	55,867 (2.73)	336,488.4 (16.46)	13.72
Registered pregnant women	61,909 (3.03)/ 63,336 (3.10)	63,336 (3.10)	43,187.6 (2.11)	0.99
Lactating mothers	45,372 (2.22)/ 45,639 (2.23)	41,095 (2.01)	39,261.46 (1.92)	-0.09

Data quality

Data quality assurance is crucial for correct interpretation, especially with the adoption of digital monitoring using indicators. In the current study, a few issues were identified concerning the quality of the data reported.

Gaps in Understanding of Data Definitions and Data Collection Methods

There was ambiguity in defining some data elements and their interpretation by those filling them. According to the definition of data elements on the AMB Dashboard, HMIS: 1.4.2 Data Element: number of PW having Hb level<11 (tested cases) (7.1-10.9) and HMIS: 1.4.3 Data Element: number of PW having Hb level<7 (tested cases), only new cases were to be reported; however, the HMIS service provider’s manual does not specify that only new cases are to be reported.

There were gaps in the interpretation and understanding of these indicators at the field level. For HMIS: 9.9 Data Element: number of children 6-59 months provided 8-10 doses (1 mL) of IFA syrup (biweekly), most facilities reported the number of IFA syrup bottles distributed among children under five years of age in the reporting month. One of the health facilities was found to be reporting the number of children given IFA syrup bottles six months back, assuming they completed biweekly IFA supplementation.

For HMIS: 1.2.4 Data Element: Number of PW given 180 IFA tablets, most facilities reported the number of pregnant females who delivered in the reporting month and were taking IFA tablets regularly. Actual pill count was not recorded at the facilities; instead, this was primarily based on the recall of the health workers.

For HMIS data elements 1.4.2 and 1.4.3, instead of the number of females with anemia and severe anemia, the number of tests with results showing anemia and severe anemia among those tested in ANC clinics each month was reported. Hence, a pregnant female who tests twice a month would contribute to two values, while a female who does not visit the facility to get tested would not be counted in either numerator. The denominator used is the number of registered pregnant women, not those tested, so the proportions obtained might be inaccurate.

The format for entering stock status indicators at the peripheral level (ANMs) required reporting of stock status as adequate or inadequate. However, none of them were sure how to assess stock adequacy.

Data Entry Errors

A validation check on HMIS data did not suggest any data entry error in the numerators. The number of pregnant women with severe anemia was less than that of those with anemia, and the number of pregnant women with severe anemia treated was less than or equal to that of those with severe anemia. However, the numerators for coverage of deworming with albendazole, as per NDD reports, were way higher than the expected beneficiaries as per AMB denominators.

Systemic Errors

Scope of capturing data by record keeping: All the data elements required in the report should be captured in the day-to-day record keeping. There were no separate NIPI registers, as suggested by AMB. At most places, there was no designated space to write the deworming status with albendazole in the second trimester of pregnancy. This was filled based on recall of the ANMs or assuming all ANCs were dewormed or as informed by ASHAs. No separate record was maintained to document children receiving 8-10 doses of syrup IFA or pregnant and lactating women receiving 180 IFA tablets.

Unavailability of guidelines and data dictionaries: No guidelines or data dictionaries were available with the frontline workers collecting, aggregating, or reporting the data. The choice of denominator being used for the calculation of the percentage of children 6-59 months provided 8-10 doses (1 mL) of IFA syrup (biweekly) was incorrect. AMB describes the denominator as the number of children 6-59 months in the district according to the census. However, the number of live births was the denominator used to calculate the indicator at the district level.

## Discussion

The AMB program was in the process of being rolled out in the district at the time of the study's conduct. In Haryana, Assuring Total Anemia Limit (ATAL) Abhiyaan was launched on 8 March 2019 by implementing the 6 x 6 x 6 strategy based on AMB. The district's anemia control efforts primarily focused on prophylactic IFA supplementation, deworming, testing, and point-of-care treatment of anemia. The coverage of IFA prophylaxis in children 6-59 months by IFA syrup distribution was 22.5%, while, in adolescents, weekly IFA supplementation in schools was reported as 85.1%. The percentage of pregnant women completing 180 days of IFA tablets was as low as 33.1%. WIFS JUNIOR was not a part of the supplementation activities occurring in Haryana. However, with the launch of ATAL Abhiyaan in Haryana, the procurement of WIFS JUNIOR for supplementation of IFA among children 5-9 years was also underway. Although HMIS includes data elements designed to indicate the stock status of IFA formulations, these remained unreported. Despite stockouts being recognized as a significant barrier to the success of anemia control measures, stock indicators in HMIS were not utilized to monitor supply adequacy in the district. The Consolidated AMB index for the district was 35.2, indicating that the performance of measures taken to control anemia in the district was unsatisfactory. The district had identified a person, the District Monitoring and Evaluation Officer, to compile and manage data reported by HMIS; however, he was not trained to analyze the reported indicators and draw conclusions about the district's performance. Issues in data quality identified were gaps in understanding data definitions by data collectors, unavailability of data dictionaries, deficiencies in the record-keeping system to capture data required to report routine indicators, and the lack of clarity in the denominator to be used.

The coverage of IFA prophylaxis among 6-59 months of children, although low (22.5%), was higher than in Haryana (3.0%) and India (8.3%) [17]. This might be due to the district's emphasis on micronutrient deficiencies addressed under the Micronutrient Supplementation Programme run by the Government of Haryana (National Health Mission) [18]. In adolescents given weekly IFA supplementation in schools, coverage was reported to be 85.1 %. This was way higher than that of the state of Haryana (51.9%) and better than the national average (28.0%) [17]. However, a significant discrepancy was observed between the denominators given by AMB and the calculated census estimates for the two beneficiary groups of children 5-9 years and adolescents 10-19 years. This discrepancy may have led to overestimating coverage in Faridabad; however, the values might still be comparable to the reported state and national averages since they were calculated using AMB denominators. The Faridabad district performed well in deworming with albendazole across all beneficiary groups. This could be due to the reporting of the population dewormed during National Deworming Day, a focused biannual activity that is well-planned and monitored during its implementation to ensure better coverage. The percentage of pregnant women completing a 180-day course of IFA tablets was 33.1%, indicating poor performance compared to the state (59.5%) and the national average (84.8%), as reported in the AMB Dashboard [17]. This was similar to the percentage of pregnant women who received IFA tablets for 180 days or more (32.0%), as reported by NFHS-5 (2021). This indicates that the district falls short of successfully implementing the long-running strategy of IFA supplementation among pregnant women.

The district's AMB score (35.2) was slightly higher than that of Haryana (28.6) and similar to the national average (34.0) for the same period. The poorer performance in prophylactic IFA supplementation among pregnant females than the other two is compensated by the IFA coverage among adolescents. The discrepancy in the denominator calculation for the adolescent age group might be falsely overestimating the coverage indicator values; this underscores the urgent need to relook into the accuracy of available denominators.

The Haryana state has a computerized HMIS, which is time-conserving and cost-effective based on previous studies [19,20]. As HMIS plays a crucial role in ensuring a continuous flow of good-quality disaggregated data on the health of populations and healthcare services [21], this ensured easy accessibility and timely data availability for decision-makers at the district level to assist in local planning, program implementation, management, monitoring, and evaluation.

On using AMB-HMIS denominators, the indicators for pregnant and lactating females were comparable with those reported by the district. This might be due to accurate reporting of denominator values in the HMIS since IFA supplementation among pregnant and lactating females has been implemented since the inception of control programs for anemia. At the same time, the other indicators are comparatively newer, and the health system has less experience implementing and reporting the same. However, despite IFA supplementation being the country's critical strategy of anemia control for years, there is still a long path to achieving 100% coverage. The AMB HMIS indicators identified for monitoring by the program did not include the domain of diagnostic services available for anemia. Despite the availability of the data element 1.4.1 in HMIS giving the number of pregnant women tested for Hb 4 or more than four times for respective ANCs (67.2% for the district under study), it was not included in the AMB HMIS indicator list.

Misreporting and mismatch of data at the subcentre level and that being reported in HMIS have been identified in a few studies before [22,23]. However, the understanding of the data collectors regarding the data they provide has often been overlooked. The strength of this study was its emphasis on identifying gaps in this understanding, which could hinder an accurate representation of frontline workers' activities in the district. Inaccurate understanding of data definitions by data collectors can lead to misinterpretation and misreporting, which may compromise the accuracy of the data and the overall evaluation of the district's performance in anemia control. This study would also serve as a baseline for assessing the performance of the AMB program strategy in the future.

A limitation of the current study was the lack of cross-verification of data reported by various health facilities in the HMIS to detect potential intentional misreporting. The study's primary aim was to provide a snapshot of Faridabad district's performance in anemia control by focusing on the AMB HMIS indicators at the district level, understanding the processes behind them, and examining the data collectors' comprehension of the data elements to ensure a valid interpretation.

## Conclusions

The AMB program was in the process of being rolled out in the district of Faridabad. On assessing the status of anemia control measures, based on the indicators reported in HMIS for district Faridabad, it could be concluded that the district's performance was unsatisfactory (AMB score: 35.2). Issues in the quality of data reported, such as the non-availability of data definitions at the field level, decreased the accuracy of data due to gaps in understanding of the data collectors and those reporting the same.

For effective monitoring, refresher training to reorient the data collectors to their role, the availability of data dictionaries, and training of district monitoring and evaluation officers to analyze and draw meaningful conclusions from the indicators can be adopted. Periodic evaluations of anemia control measures would be instrumental in identifying and eliminating barriers to implementing program strategies and achieving the ambitious targets set by the AMB program.
